# “I Ran to Make a Point”: Predicting and Preventing Youth Runaway from Foster Care

**DOI:** 10.1007/s10560-023-00930-3

**Published:** 2023-06-07

**Authors:** Kaela Byers, Jared Barton, Whitney Grube, Jessica Wesley, Becci A. Akin, Emily Hermesch, Erin Felzke, Rachelle Roosevelt

**Affiliations:** 1 School of Social Welfare, University of Kansas, 1545 Lilac Lane, Lawrence, KS 66045 USA; 2 TFI Family Services, Inc, 217 Southeast 4th Street, Topeka, KS 66603 USA

**Keywords:** Youth, Runaway, Foster Care, Shared Parenting, Family Connection

## Abstract

Youth who run away from foster care experience danger to health and safety and increased risk of adverse child welfare outcomes. By applying a concurrent mixed-methods approach, this study aimed to develop a deeper understanding of runaway risk that used a person-centered lens and amplified youth voices. Collectively, this approach can inform service innovations to support youth placed in out-of-home care. Working with a foster care agency in Kansas, data sources comprised administrative data for youth ages 12 + in care, and interview data with 20 youth, 12 + in care. Quantitative analyses involved latent class analysis followed by multinomial logistic regression to investigate whether the population of youth in care was comprised of subpopulations with differential runaway risk and whether subpopulations would predict runaway behaviors. Qualitative analyses applied modified analytic inductive thematic analysis to explore critical life experiences that may act as risk or protective factors of running away from care. Results revealed four sub-populations which were characterized by their previous family and system experiences. Additionally, class membership, gender, number of siblings, and age were statistically significant predictors of runway behaviors. Youth interviews revealed five key themes on life experiences that mitigate or exacerbate youths’ runaway behaviors. Recommendations resulting from this study were provided in three key areas: (1) improving family visitation and maintaining youth connections with self-identified family and non-relative kin; (2) supporting service approaches for youth that honor and amplify their voices, choices, and family connections; and (3) improving placement quality and individualization of services.

## Introduction

Much attention has been given to the study of placement instability in foster care. However, federal measurement of placement instability does not account for many runaway episodes, thus undercounting this important issue that exemplifies placement instability. Running away from foster care has been a longstanding and intractable problem (Nesmith, 2006). A recent ten-year study of youth running away from foster care reaffirmed known predictors based on youth and case characteristics (Branscum & Richards, 2022). Despite the attention this problem has warranted and the stability of the predictors over time (e.g., race, gender, age, reason for removal, child behavior problems, placement instability), youth continue to experience environments in foster care and family separation that prompt decisions to run away (Branscum & Richards, 2022).

Two issues suggest the time is right to re-examine youth runaway from foster care. First, advances in quantitative analyses have made person-centered modeling a standard approach over variable-centered modeling for examining complex and nuanced issues such as youth runaway, particularly among populations with high heterogeneity such as the foster care population. Variable centered analysis offers information about the relationship between variables of interest, while person-centered methods identify subgroups based on their similarities, thus “unmixing” heterogeneous populations to understand the unique nuance of subpopulations (Bergman & Magnusson, 1997). This approach allows for understanding differences of subgroups so programs and services can be targeted rather than general, and account for group-based needs and characteristics. This is particularly salient among small groups such as youth experiencing complex risks whose unique variation and needs may be washed out in analyses focusing on changes in population means. Second, while scholars have advocated for youth voice in foster care research for nearly two decades (Unrau, 2007), more active efforts are needed to include youth and young adults in current activities to re-vision and reform child welfare systems to be more responsive to longstanding problems (Children’s Bureau, 2019; Child Welfare Information Gateway, 2021). This decision in analytic method selection is methodological in terms of triangulating findings through multiple data sources. But more importantly, this approach reflects efforts to mitigate the larger social problem of program design and implementation in social services devoid of the perspectives of those most impacted – the youth themselves. The lack of youth voice where decisions are made often results in programs and policies that are misaligned with youth needs and goals (Children’s Bureau, 2019; Hyde & Kammerer 2009; Spencer, 2007; Perlmutter, 2017; Doucet et al., 2021).

This study aims to add depth to the examination of youth runaway episodes and the predictive risk and protective factors associated with decisions to run away (e.g., connection to family and loved ones, child welfare system constraints, maltreatment, nurturance, etc.). We aim to advance this issue by advancing the research with person-centered analyses and centering the voices of youth in understanding their critical life experiences. These critical experiences, which may include experiences of maltreatment, neglect, death or separation from a loved one, oppression and bias, individual or family health issues, etc., may impact future decision-making, particularly related to running away from foster care. Thus, the inclusion of qualitative methods help to contextualize and supplement quantitative findings from the perspective of youth. Further, we aimed to identify recommendations endorsed by youth to support youth-centered system transformations.

## Background and Problem Statement

### Youth Runaway Prevalence

According to recent data from the Kansas Department for Children and Families (DCF, 2022), of the 6,490 children in out-of-home care as of February 2022, 81 (1.24%) were reported as runaway. This closely mirrors the national average with the most recent Adoption and Foster Care Analysis and Reporting System (AFCARS) report published by the Administration for Children and Family (ACF) showing that approximately 1% of children in care in the United States are currently in runaway status (Children’s Bureau, 2021). A Kansas study of a one-year entry cohort from state fiscal year 2006 showed that 9.3% of children and youth had experienced a runaway incident during the study’s 30-month observation period (Akin et al., 2011). This study also found that children and youth who had any runaway incident while in foster care were significantly less likely to achieve any of the different types of permanency (i.e., reunification, guardianship, or adoption) (Akin et al., 2011).

Prevalence estimates are influenced by the duration of the observation period and the age of children and youth included in the data. Since runaway incidents occur at higher rates among adolescents, ages 12–18 (Courtney et al., 2005; Courtney & Zinn, 2009), and because runaway may be a recurrent event, reports can undercount runaway prevalence. Undercounting occurs by including all ages in runaway counts, estimating with narrowly-defined point-in-time data, such as a single day of the year, and by excluding runaway absences from foster care less than 24 h. While prior reports show children who are in runaway status at any given time, they do not capture the percentage of kids who run away at *any* point while they are in state custody and then later return to care; nor do they capture children who run away more than once. Previous studies have suggested prevalence rates for older youth range between 14 and 44%; however, some studies with higher prevalence rates focused solely on youth in specialized or residential care (Wulczyn, 2020).

### Contributing Factors

Multiple studies suggest that key youth characteristics and specific contextual factors may contribute to youth running away from foster care. Previous studies examining youth runaway show that age plays a significant role in runaway risk. Older children are much more likely to run from out-of-home care (Kim et al., 2015; Courtney & Wong, 1996; Courtney & Zinn, 2009; Courtney et al., 2005; Wulczyn, 2020; Lin, 2012; Nesmith, 2006; Nystrom et al., 2022) with studies showing that each one-year increase in age boosts the likelihood of running by 18% (Nesmith, 2006) to 58% (Lin, 2012). Approximately 90% of children who run from foster care are between the ages of 12–18 years (Courtney et al., 2005; Courtney & Zinn, 2009). The child’s age at first removal into foster care is also correlated with runaway risk. Children who run away from out-of-home care have an average age of 11 at first removal, compared to an average first removal age of 6 years for youth with no instances of running away (Lin, 2012; Courtney & Zinn, 2009; Courtney et al., 2005). Research has shown that for each one-year increase in age at first removal to out-of-home care, the odds of running away increases by 4% (Lin, 2012).

In addition to age, existing studies have also reported that females and Black or Hispanic youth are more likely to run away from foster care (Courtney et al., 2005; Wulczyn, 2020; Lin, 2012; Nystrom et al., 2022). While Lin (2012) found that children of color regardless of race were more likely to run than White children, the results of other studies examining race are mixed. Nesmith (2006) found that Native American children were more than twice as likely as White children to run away but found no other racial differences. Furthermore, Kim et al. (2015) found that race had no effect when accounting for variations within populations of counties served. Courtney et al. (2005) also noted that race had no relation to likelihood of multiple runs.

Specific contextual factors may also contribute to youth running away from foster care. Youth are most likely to run within the first 6 months of entering out-of-home care (Pergamit & Ernst, 2011; Courtney & Zinn, 2009), and one-in-five youth run within 30 days of reentering care (Courtney et al., 2005). Moreover, a history of a previous runaway episode may significantly increase the odds of a youth running away again (Nesmith, 2006; Courtney & Zinn, 2009; Courtney et al., 2005). Children in foster care with a previous runway history are 92% more likely than youth with no run history to run away again (Nesmith, 2006). Youth who had issues with substance abuse, conduct disorders, behavior problems, or mental health diagnoses were also more likely to run (Nesmith, 2006; Lin, 2012; Courtney et al., 2005). Additionally, alcohol and substance use were strongly related to increased runaway risk (Courtney & Zinn, 2009). Studies also showed a correlation between single parent families of origin and runaway risk (Lin, 2012; Kim et al., 2015).

At a systems level, placement instability and case plan goals were shown to influence runaway behavior. Youth who ran away had experienced more placements and more removals than those who did not run away (Kim et al., 2015; Pergamit & Ernst, 2011; Lin, 2012) quantified the extent to which these experiences contributed to runaway risk, as each additional out-of-home placement increased the odds of running by 4%, and each removal increased odds by 23%. One study showed youth with a permanency plan other than reunification were 89% more likely to run away (Nesmith, 2006). Many studies showed youth placed in congregate care were more likely to run (Courtney et al., 2005; Wulczyn, 2020; Courtney & Wong, 1996; Lin, 2012).

While most studies of running away from foster care have been quantitative (Bowden & Lambie, 2015), a few qualitative studies have sought to provide more descriptive information on the reasons that youth run away from foster care. Overall, the reasons may be largely categorized into “running to” family, friends, and connections, or “running from” foster care as described by Crosland and colleagues (2018). The phenomenon of “running to” is represented by qualitative studies with youth participants who indicate that they ran away due to a lack of attachment to a significant adult while in out-of-home care and were seeking to be with people with whom they have connections (Biehal et al., 2000; Finkelstein et al., 2004; Karam et al., 2013; Taylor & McQuillan, 2014). Some research has also indicated that youth ran away because the rules of foster care are new and excessively restrictive. Youth are seeking environments where they are able to have a normal teenage experience and get to do all the activities that youth not placed in foster care get to do (Crosland et al., 2018; Finkelstein et al., 2004; Taylor & McQuillan, 2014). One of these studies found that youth ran from foster care because of boredom driven by the restrictiveness of rules that exclude many youth activities (Finkelstein et al., 2004).

Regarding “running from” foster care, qualitative studies have also shown that youth ran from negative social interactions and hostile or uncaring environments (Crosland et al., 2018; Finkelstein et al., 2004; Karam et al., 2013). One study described that youth were running from environments where they had no control or say over their lives, seeking to exercise autonomy (Taylor & McQuillan, 2014). It should be noted that among these five qualitative studies, youth were participants in all of them. However, only two of the studies included participants who were exclusively youth (i.e., the other three studies included professionals and/or caregivers in addition to youth participants).

### Negative Outcomes Associated with Runaway

The risks youth experience while on the run from out-of-home care have far reaching implications for youth throughout the life course. Previous research, for example, has suggested that youth who run away from foster care are more likely to report a number of health-related issues. Specifically, youth who run away experience a higher likelihood of engaging in substance abuse (Courtney & Zinn, 2009); experiencing school issues, such as dropping out or truancy (Crosland & Dunlap, 2015; Sullivan & Knutson, 2000); being sexually exploited or trafficked (Cohen et al., 1991; Latzman et al., 2019; Yates et al., 1988); having health issues related to exposure to sexually transmitted diseases and infections (Booth et al., 1999; Courtney et al., 2005); and, experiencing an increased likelihood of attempting suicide (Yates et al., 1988).

### Study Rationale

While prior studies have illuminated many factors as contributing to youth running away from foster care, two important gaps are noted. First, few studies have examined the issue with person-centered analyses which may offer new insights to the evidence base. Second, while a handful of studies have added youth perspectives, studies have rarely integrated quantitative and qualitative data to promote both broad and deep assessment of runaway behavior in the foster care context. To move beyond identifying the universe of contributing and co-occurring factors present among youth who run away from care, we aimed to understand this issue from a person-centered quantitative lens to increase the precision of the research. Simultaneously, we sought to understand youth decisions to run away from foster care from youth perspectives through qualitative interviews with two goals. First, this approach was intended to mitigate the limitations of the administrative data and enrich and supplement the quantitative findings, rather than replicate them. Second, our goals included centering the lives, experiences, and voices of youth in out-of-home care to build authentic and honest understandings of youth running away from care and identify implications for practice that are endorsed and recommended by youth. In sum, the aim of this study is to develop a deeper understanding of runaway risk from this person-centered lens and youth-driven perspective. Findings from this study will inform program innovations to support youth placed in out-of-home care through increased placement stability and reduced incidence of runaway episodes.

With these aims, three overarching research questions guided the study.

Research Question 1 (RQ1): Is the overall population of youth ages 12 and older in foster care comprised of subpopulations of youth that are characterized by youth connections to family, youth health, critical life experiences (e.g., maltreatment, neglect, death or separation from a loved one, individual or family health issues, etc.), and youth system experiences?

Research Question 2 (RQ2): If so, does subpopulation membership predict runaway from foster care, defined as absence from their foster placement for more than 24 h?

Research Question (RQ3): From the perspective of youth, are there critical life experiences (e.g., maltreatment, neglect, death or separation from a loved one, oppression and bias, individual or family health issues, system interactions, etc.), that promote or mitigate youth decisions to run away from foster care?

## Methods

### Project Setting

The present study represents a practice-research partnership, which originated with a private foster care organization in a midwestern state – Kansas. Specifically, university researchers were engaged to conduct research after child welfare practitioners and administrators in a semi-urban and rural service area began identifying trends in the characteristics and experiences of youth with recent runaway incidents. From their casework and interviews with youth experiencing runaway from foster care, three preliminary themes emerged: (1) one or more major caregivers of youth was deceased; (2) youth lacked sibling contact; and (3) youth identified no supportive person as a social connection. To further explore and identify predictors of youth runaway from foster care, TFI Family Services, Inc. (TFI) partnered with researchers at the University of Kansas. The goal of this partnership was to conduct the present study systematically assessing the issue of youth runaway from foster care, further informing the field and ensuring youth in foster care have the resources, support, and services necessary to achieve safety and stability. The service area included in this study represents 25 counties of the state’s 105 counties.

### Study Design

The study design was a concurrent mixed method approach that included both quantitative and qualitative analyses. The goal of this design was to identify predictors of youth absence and amplify youth voices, providing insight on the factors contributing to youth decisions to run away from foster care. The study was intended to guide both the understanding of youth runaway from foster care as well as inform development of recommendations to support practice and policy improvements that promote youth stability in care. A mixed method approach was necessary to consider the factors that could be identified in quantitative data and combine that with the deeper descriptions that youth could provide from their experiences of running away. By combining quantitative and qualitative data, this study contributes richer and more complete insight to the knowledge base on runaway behavior in foster care. While our intention with the mixed methods design was to triangulate, substantiate, and strengthen all our findings, we used quantitative methods primarily to investigate RQs 1 and 2 and qualitative methods in our investigation of RQ3. All study protocols were reviewed and approved by the University of Kansas Institutional Review Board.

### Quantitative Study

#### Dataset

The quantitative investigation relied on a secondary administrative dataset exported from TFI’s case management database. The dataset included 1,127 case-level records on youth aged 12 years and older in foster care at the time of the data export (July 2021) in the agency’s Kansas service area. The dataset included case-level identifying information for linking records and de-duplication of cases, as well as demographic (e.g., race, age) and case characteristic variables that provided key insight on contextual factors affecting cases that are germane to this study. Among these case characteristics were factors related to youth connections (e.g., placement with siblings or placements within the youth’s preferred school), youth health (e.g., medical issues, developmental issues, mental health diagnoses), critical life experiences (e.g., removals, traumas, and abuse), and youth system experiences and interactions (e.g., numbers and types of out-of-home placements, number of workers, family visits). Variables were selected based on input from agency administrators and practitioners as well as from existing literature.

Some variables of interest were not available from the administrative data. Therefore, we created proxy variables that resembled the variables of interest when it was possible to do so, and supplemented with qualitative inquiry when it was not possible. Specifically, the administrative dataset contained limited information to operationalize and measure *sibling proximity, community violence*, and *sexual trauma*; thus, we created proxy variables from the dataset to approximate the variables of interest. For example, while we knew from the administrative data when youth were placed with a sibling, we were not able to ascertain the proximity of youth to siblings who were not placed in care with them. Because such a variable is essential for understanding connections to family of origin, we used a calculated variable (i.e., *preferred school*) that combined the youths’ preferred school choice, presumably in their home community, and the youths’ current placement to characterize youth proximity to family. For *community violence*, that meant youth records were designated with some form of gang involvement, and for sexual trauma, youth were identified as having been commercially exploited sexually.

Of note, history of runaway was not included as a variable in this analysis. Even though previous literature has identified this factor as important (Nesmith, 2006; Courtney & Zinn, 2009; Courtney et al., 2005), we intentionally chose not to include this variable as it was not aligned with the goal of this study. Our goal was to predict other relational and environmental risk and protective factors that may be associated with running away from a placement. Our aim with this approach was to identify characteristics and conditions early in the life of a case to head off emergent factors that may influence a youth’s to run away from a placement. ***Sample***.

Of the 1,127 youth in the secondary administrative dataset, 81% were identified as White and Non-Hispanic. Only 6% and 5% were identified as Bi-racial and Black respectively, and only a little more than 2% of the sample were identified as Hispanic. The sample contained a slightly higher proportion of males (51%) to females (48%). Youths’ mean age was 16.68 years (SD = 2.28), and on average, youth in the sample had at least one sibling (M = 1.17, SD = 1.38).

### Quantitative Analysis

A three-step analytic process guided our quantitative analysis. To support answering RQ1, we applied latent class analysis (LCA) in the first step to determine if the population of youth in out-of-home care with was comprised of subpopulations of youth characterized by youth connections, youth health, critical life experiences, and youth system experiences. LCA was chosen for its utility in identifying subpopulations (i.e., classes) contained within a larger dataset. LCA supported our person-centered approach to this study as it provides a model for describing the sample population across a set of individual characteristics and behaviors rather than describing differences among single variables (Collins & Lanza, 2010; Lanza & Cooper, 2016; Meeusen et al., 2018). Steps two and three supported the investigation into RQ2. In step two, we used multinomial logistic regression (MLR) to determine if individual characteristics—race, gender, age, and number of siblings—predicted class membership. In step three, we used a binary logistic regression (BLR) to examine if membership in classes established in step one predicted if a youth had a runaway episode. The dependent runaway episode variable was dichotomized as a “1 = Yes” or “0 = No” variable.

*Step 1: LCA.* We performed an iterative LCA process to determine the optimal number of classes that best fit the data. Variables used in the LCA included 21 categorical variables and eight continuous variables extracted from youths’ current removal episode. Table 1 contains a list and description of variables used in the LCA.

**Table 1:** Variables Used in Latent Class Analysis

Variable	Description
*Categorical Variables*	
Preferred School	Youth’s current school is equal to the youth’s preferred school
Placed w/ sibling	Youth currently placed with a sibling
Community violence	Youth has “Gang Involvement” selected under “Identified Behavioral Issues”
Developmental issues	Youth has an “Identified Developmental Issues” under “Identified Behavioral Issues”
Medical issues	Youth has an “Identified Medical Issues” under “Identified Behavioral Issues”
Sexual orientation	Youth has “LGBTQ” selected under “Identified Behavioral Issues”
Substance use issues	Youth has “Chemical Dependency” selected under “Identified Behavioral Issues”
Requires doctor or therapist	Youth requires doctor or therapist visits as selected under “Identified Behavioral Issues”
Sexual trauma	Youth has been commercially exploited sexually
Removal: Child Bx	Youth’s removal related to child’s behavior
Removal: Child disability	Youth’s removal related to child’s disability
Removal: Child Drug	Youth’s removal related to child’s drug abuse or use
Removal: Housing	Youth’s removal related to inadequate housing
Removal: Neglect	Youth’s removal for reason of neglect
Removal: Parent Death	Youth’s removal due to parental death
Removal: Incapacity	Youth’s removal due to parental incapacity
Removal: Incarceration	Youth’s removal due to parental incarceration
Removal: Physical Abuse	Youth’s removal for reason of physical abuse
Removal: Relinquishment	Youth’s removal related to relinquishment of parental rights
Removal: Sexual Abuse	Youth’s removal for reason of sexual abuse
Removal: Parent Drug	Youth’s removal related to parental drug abuse
*Continuous Variables*	
Number of placements	Total number of placement settings a youth has experienced
Average visits	Mean number of family visits a youth received monthly
Number of workers	Total number of case workers assigned to a youth
Sum of home care	Total number of home-based placements a youth has experienced
Sum of congregate care	Total number of congregate-care placements a youth has experienced
Sum of hospital stays	Total number of hospitalization episodes a youth has experienced while in out-of-home placements
Sum of incarceration	Total number of incarceration episodes a youth has experienced while in out-of-home placements
Sum of DSM diagnosis	Total number of DSM diagnoses a youth has

Using these data, we iteratively compared four sets of classes: 1) a two-class model compared to a one-class model; 3) a three-class model compared to a two-class model; 3) a four-class model compared to a three-class model; and 4) a five-class model compared to a four-class model. We stopped upon analyzing the five-class model as interpretability of class membership diminished. Model fit statistics to assess the best class-solution included Akaike’s Information Criteria (AIC), the Bayesian Information Criteria (BIC), the Vuong-Lo-Mendell-Ruben (VLMR) Likelihood Ratio Test, and the bootstrap likelihood ratio test (Collins & Lanza, 2010; Neely-Barnes, 2010). As each class was added to the model, the AIC and BIC numbers decreased indicating better model fit (Collins & Lanza, 2010; Neely-Barnes, 2010). However, the five-class model failed to replicate the best log-likelihood value. As such, the four-class model was selected as the best fitting model. Next, we used entropy scores to measure the quality of class membership. Finally, we doubled starting values for four-class model and reran the model to ensure the best log likelihood was determined.

*Step 2: MLR.* Our second quantitative analytic step involved a multinomial logistic regression (MLR) to examine whether demographic characteristics were significant predictors of class membership. As such, a variable indicating the class membership of each youth case established in step one was used as the outcome variable for the MLR. Predictor variables of interest included youths’ race, gender, age, and number of siblings. Because low numbers of youth were reported for certain race categories, we combined the race variable used in the MLR into four categories as follows: White/Non-Hispanic, Black, Bi-Racial, Other. The gender variable was coded in accordance with the youths’ reported gender identity (i.e., male, female, transgender). The age and number of siblings variables were both continuous variables.

*Step 3: Binary Logistic Regression.* Our third and final quantitative analytic step involved a binary logistic regression (BLR) to determine if class membership, as identified in step one, significantly predicted whether youth had a runaway episode from foster care, while controlling for the demographic variables used in step two.

### Qualitative Study

#### Dataset

Our qualitative inquiry examined data generated from interviews the research team conducted with 20 youth. Each interview was professionally transcribed. After transcription, two members of the research team reviewed each transcript to verify its quality and scrubbed each of any personal identifying information. Validated transcripts were stored, managed, and analyzed using Dedoose software (Version 6.2, 2016).

#### Participants

Participants for the qualitative interviews were recruited from a random selection of youth cases (n = 247) in the original secondary administrative dataset. We used purposive sampling techniques aimed at capturing a representative sample of youth with and without previous runaway episodes as well as younger youth ages 12 to 14 years and older youth ages 15 to 17 years. Youth were recruited for interviews through a four-step process. First, the research team generated a list of youth selected through the purposive randomization process. Second, TFI case managers used the list to introduce the study and obtain assent from youth to be contacted by the research team. Third, for assenting youth, TFI administrators gave the research team written consent for youth participants and youth contact information. Finally, a member of the research team contacted youth via telephone to reaffirm their assent and schedule interviews to be conducted via the youth’s preferred platform (e.g., phone, Zoom).

In total, 33 youth assented and were contacted to schedule an interview. Of those 33, one youth decided not to participate after being contacted, two did not attend scheduled interviews, and ten were not responsive to outreach. The remaining 20 youth agreed to and participated in interviews. Participants received a $50 prepaid debit card for their time.

Participating youth had a mean age of 14.7 years and an average of 1.3 siblings. Eleven youth identified as female, eight identified as male, and one identified as transgender. Seven youth had at least one previous runaway episode. Placement types for youth interviewed varied. The most common placements included nine youth in relative placements and seven in non-relative foster homes. One youth was living in a psychiatric residential treatment facility, and the remaining three youth were living in a qualified residential treatment program.

#### Data Collection

We conducted in-depth interviews with youth regarding their experiences associated with critical life events (e.g., maltreatment, neglect, death or separation from a loved one, oppression and bias, individual or family health issues, system interactions, etc.) thought to serve as risk or protective factors impacting the likelihood of running away from foster care. As such, we used a semi-structured interview guide designed specifically to generate youth responses and narratives regarding their life experiences. Interviews lasted between 19 and 80 min. Sixteen youth opted to conduct interviews over the phone and four opted for virtual video conferencing via Zoom. Interviews were conducted by two researchers, one with master’s level social work education and the other with PhD level social work education, with a specialty in youth mental health. Interviewers were trained by principal investigators of the study to conduct the interviews and conducted practice sessions in preparation for interviews to ensure a level of standardization in the data collection. Further, interview protocol included procedures for connecting youth to supportive services as needed or identified by the youth.

Three domains were examined during youth interviews to gain a deeper understanding of youths’ experiences with critical life events derived from the literature and from the preliminary analyses informing this study: (1) life history milestones and other contextual relational factors; (2) system experiences; and (3) youth recommendations for system changes to better support youth in foster care. Questions from each domain were presented to youth to generate conversation about their experiences with events and other life factors. Additional probing questions were used to elicit more in-depth information about these experiences from youth participants as needed.

Questions included in domain one included descriptions and experiences related to current connections to family, description of siblings, gender and sexual identity, and prevention service history. For example, interviewers asked youth if there were times their basic needs went unmet and to describe their neighborhood and housing. Interviewers used prompts if youth did not understand the meaning of the question. Questions in the second domain focused on system experiences including, among others, number and impressions of case managers and other workers assigned to the youths’ case, history of maltreatment reporting, and youth attitudes and perspectives of the system (e.g., “Describe your experience in the foster care system”). The second domain also addressed youth runaway behaviors asking: “have you ever ran away for more than a day?” with a prompt to have youth tell interviewers more about these experiences. Questions in the third domain inquired about youths’ recommendations for future changes in the service delivery system (e.g., “At any point, was there a service they felt like they needed, but didn’t receive? ” and “If they had a magic wand, what changes would they make to the foster care system?”).

### Qualitative Analysis

For qualitative analyses, transcripts of qualitative interviews were analyzed using a Modified Analytic Induction methodology (Bogdan & Biklen, 1998) to conduct an analysis of youth experiences and critical life experiences. Modified Analytic Induction is an emergent methodological design that allows for the examination of preconceived hypotheses identifying patterns of behavior, such as youth decisions to run away from out-of-home placement. Application of this methodological approach included examination of hypotheses that were derived from initial exploration of runaway events to confirm or iteratively revise hypotheses. Rather than seeking a universal causal hypothesis, we used this method to describe patterns of behavior that are anchored in previous research while also allowing for the emergence of new ideas (Gilgun, 1992, 1995).

Analyses occurred iteratively across stages. First, the research team systematically coded interview transcripts as a team of four coders including principal investigators and research team interviewers. The team used open coding to classify the data into individual codes. Initially, as a research team, we coded inductively without a predetermined coding structure. Then, we performed a second iteration of coding independently, ensuring the focal constructs addressed in the interview protocol were captured, and further defining additional inductive codes based on interview content (Corbin & Strauss, 2015) and checking for inter-coder reliability (Miles et al., 2014).

After coding, we applied thematic analysis (Braun & Clarke, 2012) deductively and collaboratively applying refined codes to relevant interview excerpts to identify, organize, and understand themes and patterns across the interview transcripts. Thematic analysis occurred collaboratively among our research team as we classified individual codes into themes and compared emergent themes to the a priori themes established from prior research. We also sought to identify any negative cases that may indicate need for iterative revision of the initial hypothesis. Disagreements were resolved through team discussion until consensus was achieved. Upon conclusion of initial analyses, we presented summary findings to key agency staff. We also invited all participating youth to attend a co-interpretation meeting via Zoom. Five youth who participated in interviews attended this session and engaged in in-depth discussion about additions and alternative interpretations to inform and refine findings and recommendations.

Presenting our findings to both staff and youth served multiple purposes. First, it provided a method of member checking and co-interpretation necessary to ensure trustworthiness of the findings. Second, it demonstrated our commitment to sharing power and authentically centering the voices of youth and those closest to the issue at hand. And third, it supported the discovery of erroneous interpretations, additional negative cases, and the refinement of findings and recommendations to align more closely with the intent of participant youth. Based on these discussions with staff and youth, we refined and finalized the findings, themes, and recommendations reported in this paper. Primary coding and analyses were conducted using Dedoose Analytic Software (Version 6.2) for data storage and retrieval, to organize codes, categories, and sub-categories, and create analytic memos about codes and categories and the perceived relationships between them.

## Results

### Quantitative Results

#### Latent Class Analysis

**Fig. 1:**
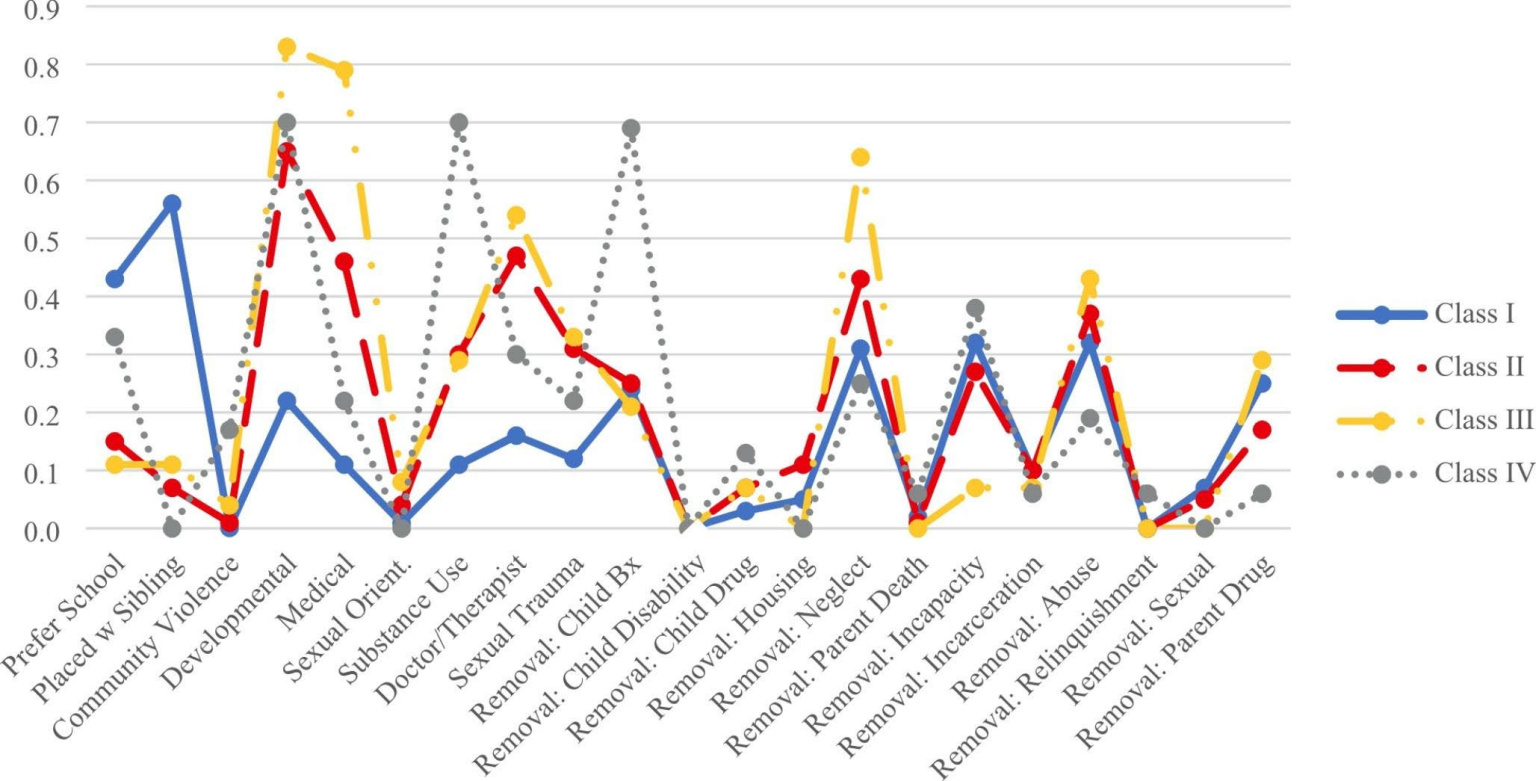
Probability of “Yes” Response for Categorical Variables

**Fig. 2:**
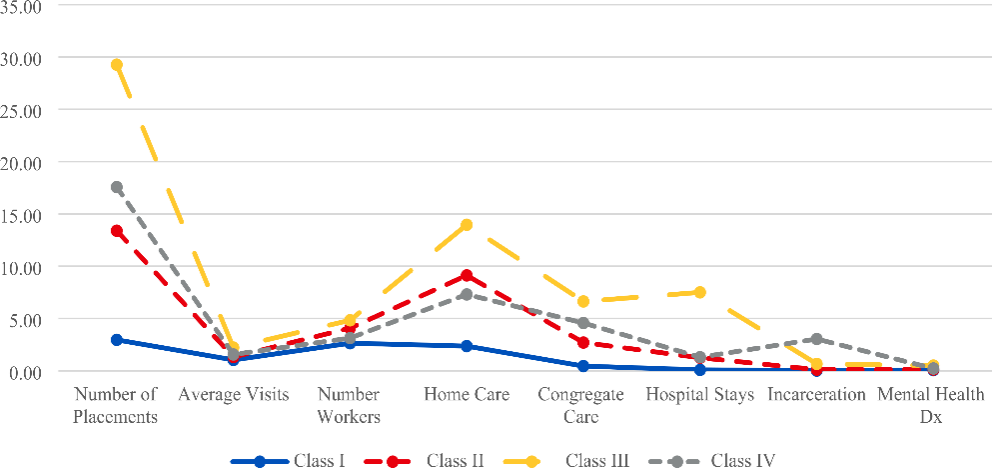
Estimated Means for Four Class Model

Results from the LCA indicated the sample used in the quantitative analysis was comprised of four distinct classes. Table 2 shows model fit statistics for all model solutions tested. For the selected four-class solution, Fig. 1 displays each class’s probability of a “yes” response for each of the categorical variables included in the model, and Fig. 2 displays each class’s estimated means for each of the continuous variables included in the model. Individual item-response probabilities for each categorical variable as well as the estimated means for each of the continuous variables included in the model are available from the authors upon request. Based on the conditional item response probabilities, estimated means, and clinical or substantive relevance of the items, the four classes were conceptualized as follows: *High Environmental Stability/High Familial and Environmental Connections* (Class I; 83%), *Moderate Connection/Moderate Environmental Risk* (Class II; 13%), *Low Environmental Stability/Low Community Connection/High Familial Connection* (Class III; 2%), and *Low Stability and Low Environmental Connection* (Class IV; 2%). Class sizes varied substantially, with the large majority of participants comprising Classes I (83%) or II (13%). Classes III and IV, comprising 2% of the sample, respectively, were retained despite their small size as they are substantively important and represent the population of youth experiencing more complex risk factors across measurement domains. The following describes how we interpreted each of these classes.

**Table 2:** LCA Model Fit Statistics

	AIC	BIC	Entropy	VLMR	Adj-LRT
1 class	46191.85	46377.86	--	--	--
2 class	43297.96	43634.79	0.98	0.05	0.05
3 class	42007.20	42494.85	0.97	0.24	0.24
4 class	41039.75	41678.22	0.98	0.30	0.30
5 class	40194.15	40983.45	0.98	0.75	0.75

#### Class I: High Environmental Stability/High Familial and Environmental Connections

Class I was the largest class and was conceptualized as the *High Environmental Stability/High Familial and Environmental Connections* class. Close to 83% of the sample was estimated to be a member of Class I. Primary characteristics of this class were low system involvement (i.e., few number of placements) and these youth had the highest probability of being in their preferred school environment and being placed with their sibling(s) compared to the youth in other classes. These youth also had low probabilities regarding having identified medical or behavioral issues. The average age for this class was approximately 16.5 years and was almost evenly split in terms of males and females.

#### Class II: Moderate Connection/Moderate Environmental Risk

Class II was the second largest class, as 13% of the sample were estimated to be members of this subgroup. Class II was conceptualized as the *Moderate Connection/Moderate Environmental Risk* class. Youth in this class had moderate to high system involvement. On average, youth in this class experienced close to 14 placements, with most placements in home-based settings rather than institutional or congregate care settings. Youth in Class II had the highest probability of being removed from their parents’ care due to inadequate housing compared to youth in other classes. Youth in Class II also had moderate to high probability of being removed due to neglect concerns. Other characterizations of the youth experiences in this class include moderate to low connection with their preferred school environment and siblings, and moderate probability of having identified medical concerns.

#### Class III: Low Environmental Stability/Low Community Connection but High Familial Connection

Class III, comprising 2% of the sample and labeled *Low Environmental Stability/Low Community Connection but High Familial Connection*, was primarily categorized by the high number of placements estimated for each youth. Youth in Class III were estimated as having close to 30 placements. Additionally, they had low probability of being in their preferred school, and high probability of developmental, medical, and mental health issues. Furthermore, youth in this class were estimated as having high instances of placements in acute psychiatric facilities. These youth comprised more females than males and had higher probabilities than other classes of being removed due to parent and parenting related reasons (i.e., parental substance use, physical abuse, and neglect). However, these youth also had the highest estimated mean family visits per month, compared to other classes. Youth in this class had 2.2 family visits per month, which though still small is more than double the mean number of visits for youth in Class I.

#### Class IV: Low Stability and Low Environmental Connection

Based on the item response probabilities and estimated means for this class, researchers characterized youth in Class IV as the *Low Stability and Low Environmental Connection Class*. Youth in this class, representing 2% of the sample was primarily characterized by having higher juvenile justice involvement, high instances of identified substance use issues and mental health concerns. Additionally, youth in this class had the highest probability of having a parent be deceased or incarcerated, indicating low family stability. Furthermore, youth in this class tended to be older male youth and, though small, was the most racially diverse group. Youth in Class IV were more likely to be removed due to child behaviors and youth in this class had the fewest number of siblings. Likewise, they were the least likely to be placed with siblings.

### Multinomial Logistic Regression

After the LCA, researchers conducted a multinomial logistic regression to determine if youth demographic characteristics (i.e., race, gender, age, and number of siblings) were significant predictors of class membership. Prior to analyzing the results of the regression model, model fit indices were obtained. Table 3 contains descriptive information for each class.

**Table 3:** Descriptives for predictor variables by class membership

Variable	Class 1		Class 2		Class 3		Class 4	
	n	%	n	%	n	%	n	%
*Categorical*								
Gender Male	485	51.7	10	41.7	72	51.1	16	69.6
Female	454	48.3	14	58.3	69	48.9	7	30.4
Race/Ethnicity White/Non-Hispanic	755	84.5	22	91.7	115	83.9	16	72.7
Black	43	4.8	2	8.3	11	8.0	1	4.5
Hispanic	23	2.6	0	0	2	1.5	1	4.5
Bi-Racial	55	6.2	0	0	9	6.6	4	18.2
Other	18	2.0	0	0	0	0	0	0
*Continuous*	Mean (SD)		Mean (SD)		Mean (SD)		Mean (SD)	
Age	16.57 (2.32)		17.26 (2.45)		17.08 (2.04)		17.88 1.22)	
Number of siblings	1.17 (1.37)		1.04 (1.23)		1.26 (1.53)		0.52 (0.73)	

Based on the likelihood ratio (LR) test, the model containing the demographic predictor variables of race, age, gender, and number of siblings, represents a significant improvement in fit relative to a null model or a model with no predictors [LR _*X*_^*2*^(21) = 40.06, p < .05]. In addition to the LR test, the McFadden r-squared value was also obtained. Based on the McFadden’s r-squared value, the full model containing the predictor variables represented a 3.2% improvement in fit relative to the null model. Results from the goodness of fit indices revealed non-significance, which provide further evidence of a well-fitting model.

After the model fit indices were obtained, the nominal variable of class membership, with four categories, was regressed on four predictor variables including race, gender, age, and number of siblings. Results from the MLR model indicated age and the child or adolescent’s number of siblings as statistically significant predictors for some class comparisons. Specifically, regarding Class I, results revealed that for every one-year increase in a child or adolescent’s age, the odds of being in Class I compared to Class IV decreased by 20% (*OR* = 0.80, *p* = .03, 95% CI [0.65 –0.98]). Furthermore, results revealed that for each additional sibling a child or adolescent had, they were 60% more likely to be in Class II than Class IV (*OR* = 1.60, *p* = .05, 95% CI [0.99 − 2.57]). Table 4 details the MLR results.

**Table 4:** Odds Ratios and Confidence Intervals (Cis) from Multinomial Logistic Regression (MLR), Regressing Class Membership on Demographic Variables

	Odds Ratio [95% Confidence Interval]		
Predictor Variables	Class I. n = 939	Class II. N = 141	Class III. N = 24
Male	0.46 [0.18, 1.16]	0.47 [0.18, 1.24]	0.31 [0.09, 1.04]
Age	0.80 [0.65, 0.98]*	0.88 [0.71, 1.10]	0.91 [0.69. 1.19]
White/Non-Hispanic	1.79 [0.0, 0.0]	1.91 [0.52, 7.08]	1.99 [0.0, 0.0]
Black	1.77 [0.0, 0.0]	3.06 [0.28, 33.11]	3.06 [0.0, 0.0]
Bi-Racial	4.64 [0.0, 0.0]	0.54, [0.54, 0.54]	5.02 [0.0, 0.0]
Num of Siblings	1.47 [0.18, 1.15]	1.60 [0.99, 2.57]*	1.42 [0.81, 2.46]

Note. All class and variable Ors are compared to Class IV (n = 24); Females served as the reference group; Other served as the race reference group

* p < .05

### Binary Logistic Regression

To answer RQ2, a binary logistic regression (BLR) was conducted to determine if class membership could predict runaway behaviors. Descriptive analysis indicated that close to 21% (n = 233) of the sample had engaged in a previous runaway, while 79% (n = 893) had not. Runaway behavior was regressed on class membership, gender, number of siblings, age, and race. Results from the BLR revealed that class membership characterized by youth experiences, gender, number of siblings, and age were all significant predictors of runway behaviors.

Regarding class membership, youth in Class I (*High Environmental Stability/High Familial and Environmental Connections*) were close to 98% *less* likely (OR = 0.018, p < .001, 95% CI [0.004, 0.081]) to have a runaway than youth in Class IV (*Low Stability and Low Environmental Connection*), while youth in Class II were 83% less likely (OR = 0.19, p < .05, 95% CI [0.042, 0.874]) to have a runaway than youth in Class IV. Additionally, for every sibling a youth was reported as having, they were 15% *less* likely to have a runaway episode (OR = 0.85, p = .02, 95% CI [0.74, 0.98]).

Regarding gender, males were 62% *more* likely (OR = 1.62, p = .007, 95% CI [1.14, 2.32]) to have engaged in a runway episode. Finally, for every year increase in a youth’s age, they were 29% more likely to have a runaway episode (OR = 1.29, p < .001, 95% CI [1.19, 1.40]). See Table 5 for complete BLR regression results.

**Table 5:** Binary Logistic Regression (BLR) Results from Regressing Class Membership on Youth Absence Outcome

Predictor	*p*	*e* ^β^	*CI*
Class I	< 0.001***	0.018	[0.004, 0.081]
Class II	0.03*	0.19	[0.042, 0.874]
Class III	0.40	0.47	[0.080, 2.72]
Gender (Male)	0.007**	1.62	[1.14, 2.32]
Number of Sibs	0.02*	0.853	[0.74, 0.98]
Age	< 0.001***	1.29	[1.19, 1.40]
Black	0.12	1.73	[0.865, 3.47]
Bi-Racial	0.55	1.24	[0.611, 2.51]
Constant	< 0.001	0.003	

Note. The following served as reference groups: Class IV; Females; White/Non-Hispanics

* p < .05 ** p < .01 *** p < .001

### Qualitative Findings

Interviews conducted with youth yielded five primary themes supported by numerous subthemes and illustrative quotes. Emergent themes illustrate the common experiences, milestones, and events, along with widespread perspectives of youth related to their families of origin, their circumstances in foster care and with the system, and their decisions to either runaway or stay in a placement. Key themes are presented in depth in this section.

#### Youth Described the that Losing Family Connections Left them with Fewer Resources and Increased Instability

Youth described profound connections to extended families of origin, which included connections with relatives, kin, and non-relative kin. These connections were often characterized by positive regard, deep bonds, and remembrance of positive core memories, mixed with complex experiences of poverty, hardship, abuse, neglect, and uncertainty. Despite these complex and opposing experiences and feelings, youth largely described the importance of these connections. In recounting critical life experiences, many youth described complex and multigenerational experiences of abuse and neglect, co-occurring with instances of parental substance use, harsh discipline, instability, and periods of absence from parents related to incarceration, extended drug use, court orders preventing contact, abandonment, and custody/relationship disputes. Many youth expressed clear understanding of reasons the child welfare system intervened to ensure their safety. For example, one youth shared that before entering foster care “I was living with my mother and my father…it wasn’t the most, you know, happy situation, because my father was an alcoholic and he was always pretty abusive.”

However, despite consistent reports of complex adversity, youth routinely described relationships and bonds with members of their families of origin as both positive and important to them, even when they knew, understood, and accepted they would not return home. One youth described how his relationship with his stepfather was characterized by both abuse and trust:

My stepfather was abusive, but I mean most of the things he did, even if his methods were extreme, were because of … I mean he did everything he could to try and help us and everything. I’ll admit myself that a lot of the decisions he made were not the best, but he was still there with all that in times when it counted.

Another youth described gratitude for his mother’s efforts to care for him despite the hardship they experienced as a family, stating I didn’t have like the best childhood growing up… we really had basically no money, but every day, I would thank my mom because she’s working hard,” reflecting the complex and sometimes mixed emotions youth experienced.

Other youth described important and supportive relationships with extended family such as a grandfather who one youth appreciated for “actually taking care of me and actually letting me do things,” and an uncle who was “really cool” and taught the youth “how to draw, how to get started on poetry and stories, like he was.” This youth described his uncle as “mostly my teacher on everything.” In some cases, these extended relationships were maintained over time and could serve as permanency resources for the youth. One youth reported that he did not want to “go back with my mom. My plan is not actually trying to go with my mom, I’m actually trying to go with my uncle on my dad’s side.” Despite knowing his best and safest option was not to live with his mother permanently, this youth acknowledged that severing the relationship completely may not be the best option.

Beyond simple appreciation and the tangible support of these relationships, youth valued them to such an extent that they described willingness to sacrifice environmental and physical safety to maintain family bonds and attachments, both prior to removal *and* while in out-of-home placement. This manifested in multiple ways, with some youth being willing to endure difficult, risky, and in many cases very dangerous conditions to stay with family, and second, youth willingness to run away from foster care in an effort to maintain relationships they identified as important. In one case, the youth described working together as a family unit with his family of origin to prioritize basic needs, which supported staying together prior to their removal from the home. In another case, a youth with significant runaway history described a particular runaway incident in which he turned himself in upon assurance that placement with his mother would be considered. The youth stated “That’s the only reason I came back [to be with mom]. If I, if I hadn’t had that option then I never would have came back.”

In yet another case, the youth and the system had disparate views regarding suitable living conditions for placements. This youth described how a potential relative placement they saw as viable, supportive, family-centered, and willing to serve as a placement, was deemed not allowable by the child placing agency due to caregiver behaviors. This decision was made despite there being no offense, by state or federal policy, that would prohibit relative placements. In this case, the youth valued the potential support inherent in this placement with their “favorite grandpa and grandma”, despite potentially risky activity in the home (i.e., marijuana use by members of the family), while the system ultimately saw only risk and liability.

Though other issues unknown to the youth may factor into this specific scenario, it is important to note that this issue may also reveal a potential cultural, medical, and religious gap in child welfare system practice. Strict interpretation of policy that only outlines that a home “is environmentally and psychologically safe for children” and does not otherwise specify rationale or approval criteria results in practice decisions that may be inappropriately restrictive and inconsistent. For example, in this case a relative placement was ruled out, seemingly without regard for medical, spiritual, or cultural practices that include use of marijuana and may thus disregard legitimate practices of youth and their families. This approach serves as a mechanism supporting the best interest of the state and child placing agency (i.e., liability and risk management), potentially over the needs and best interests of youth (i.e., family connection and stability).

Finally, the deep importance of family to youth was manifested as a protective factor supporting family stability prior to removal when youth had access to these supports. For example, one youth shared that “it was no like, rainbows and unicorns…as long as I was with my family you know, things are tolerable.” However, disconnection from these bonds left youth with fewer family resources and served as a destabilizing factor. One youth shared how an historically supportive relative whose presence may have mitigated future adversity died, leaving a tremendous support gap for this youth:

After I turned 12, and later on, he [uncle] was gonna adopt me and everything because he saw how bad and mistreated I was. And then he found out he had stage four lung cancer and liver cancer, and so we didn’t have much time left with him. He died after my birthday.

In fact, nearly half of youth interviewed (n = 8) reported death of key familial figures before or during their placement into foster care, including grandparents, siblings, parents, stepparents, and step grandparents, leaving a dearth of meaningful connections for youth. At least two youth also experienced separation from a caregiver due to incarceration, and two youth reported actual or perceived abandonment, further reducing their resources and connections.

#### Family Bonds and other Relationships Associated with Meeting Basic Needs while in Care were Strong Protective Factors Influencing Youth Decisions to Run Away or Return

A second theme closely related to the first theme emerged, with youth identifying key factors influencing their decisions to return to care or remain absent during runaway episodes. These factors involved both physical and concrete needs as well as family relational factors as motivators of youths’ decisions. One youth identified a basic health need they could not access on their own as the primary motivator for returning to care, noting that they only came back for their asthma inhaler. Other youth reported lack of basic resources (e.g., shelter) as reasons for returning, with one youth reporting “it was way too hot, and I just kind of knew I screwed up,” and another youth sharing their “didn’t really have anybody to go to” and ended up trying to “sleep and hide under a bush.”

When it came to the influence of maintaining bonds with, caring for, and protecting family members, youth were much more descriptive. At least two youth who ran away from foster care expressed a perceived need to care for or protect loved ones as motivating their decision to return. One youth described being a mother herself and that returning to foster care after multiple runaway episodes was necessary to maintain bonds and custody of her child, and another youth perceived returning to care after a runaway episode as a means for setting a positive example for their siblings:

I didn’t want my brothers to see me like that. I’m the older sibling. I’m supposed to be the good role model, and I felt like if I continued to do the stuff that I was doing, or I didn’t come back, they wouldn’t really have anybody to look up to.

As in this example, kinship bonds appeared to play a role in youths’ decisions to run away and their ability to remain absent from foster care. More specifically, youth described seeking out non-relative kin relationships with external caregivers that emulated parenting relationships and familial bonds to fill needs that were lacking in foster care. While the details of how youth were able to form these relationships are vague, an account from one youth explained how an opportunity for a relationship like this influenced their decision to run away from foster care: “I knew somebody that they would take care of me. I moved to their home with them [and] they took care of me.” Two youth reported seeking out and leveraging old friendships and other adult caregivers for support. One youth reported friends “let me stay because like, their mom wanted to make sure I was safe.” And another youth reported having “a couple of friends that I grew up with…I knew his parents would take me in.” Leaving those bonds behind was also a barrier to returning to foster care. One youth shared that any willingness to return was tempered by fear of losing key relationships: “After a while like I only considered going back into the state once, but that would involve me leaving my friends behind, so it wasn’t the most, it wasn’t the best thing that I was willing to do.” Connections with family and kin were important to all youth interviewed and emerged as a primary factor in runaway decision making.

#### Fear and/or Lack of Control of Own Circumstances Perpetuated by System and/or Placement Constraints Increased Risk of Runaway Decisions

A third emergent theme surfaced as youth shared stories of system and placement constraints that limited youths’ sense of control and increased youth fear around life circumstances. Together, these constraints and the resulting feelings and experiences may have contributed to youths’ decisions around running away from foster care. The loss of family relationships and bonds generated considerable fear and served as a primary motivator of youths’ decisions. One foster youth parenting their own child described how a case worker would leverage their relationship with their baby as a means of controlling the youths’ decisions. The youth reported the case worker telling her they “wanted to take the baby or turn me in…” and that these conversations “…really affected me a lot.”

In addition to fears of losing key relationships, youth described how uncertainty and a lack of clarity regarding foster care placement standards and norms, and discomfort with restrictive rules led them to experience further emotional traumas and feel powerless. Youth explained how these experiences and constraints motivated their decision-making. One youth indicated that caregivers’ volatile reactions at one placement induced anxiety that provoked the youth’s flight response: “I would tell them, ‘Hey, please don’t scream.’ That was a really anxiety inducing thing. And they would scream or yell, and I’d get triggered and I would run away.”

Another youth offered insight into how restrictive rules in a particular placement combined with disengaged foster parents contributed to their decision to run away. The youth elaborated:

They didn’t really pay attention to me… so, I kind of wanted to do my own thing [but] I was never able to leave. I wasn’t able to go to the store. I wasn’t able to chill with friends. I wasn’t able to do anything. So, I ran away because I wanted my freedom back.

In the same narrative, the youth offered further insight into the tradeoffs associated with regaining some freedom and control – their basic needs going unmet.

So, when I [ran away], I did get all the freedom that I wanted, but I didn’t get the reassurance of going back to the same place… having a bed and having something to eat at night or having clothes to wear or just having a roof.

These examples highlight the need for clarity and developmentally appropriate autonomy for youth in foster care as a means of mitigating runaway behavior.

Other youth described how the restrictions placed on them simply due to their state custody status highlighted their separateness and contributed to feeling othered and shamed. Consistently, youth were able to articulate the items, privileges, and experiences they were not allowed to have. This issue is best illustrated in one seemingly small detail, shared over and over again in interviews with youth – trampolines. One youth shared “I want a trampoline…it’s just a foster care rule, like, we’re not allowed to have them…I guess they don’t want to be like sued or whatever.” And when asked what they would change about foster care, another youth stated “every kid gets a trampoline,” illustrating this persistent desire for normalcy in their experiences. Other restrictions noted included access to cell phones, sleepovers, and staying home alone – all milestones and rites of passage generally available to youth not in foster care. These restrictions, which appear based in reducing liability for the state, disregard the developmental and social-emotional needs of youth and undermine the parental decision-making of foster parents who are otherwise trained, licensed, and trusted with the care of youth. Further, these restrictions are seen by youth as unfairly punitive, drawing attention to the distinction of these youth as different and adding to the frustration and lack of belonging experienced by youth that may prompt runaway episodes.

Finally, runaway decisions were sometimes driven by low impulse control typical in an adolescent population. In these cases, youth made rash decisions when faced with constraints they felt were unreasonable, behavior typical of their developmental stage. One youth stated “it [running away] was a dumb decision, uh, I got caught up in everything in anxiety and everything, and I was like, “Hey, can you come and get me and they’re like, “Yeah, sure.” So they came and got me.” However, fear and powerlessness were also evidenced in decisions to return to care from a runaway episode. When asked about returning to foster care after running away, many youth described letting themselves get caught by dropping hints to key contacts and staying in locations where they were likely to be found. This seemed to reflect two distinct reactions. First, some youth expressed resignation to the inevitability of being found and punished, reflecting an overall feeling of powerlessness. In other cases, “letting” themselves get caught, or running in the first place, signaled youth attempts to assert some personal agency in what they viewed as an overly restrictive environment.

#### Supportive Placements Extending Beyond Basic Needs and Promoting Belonging and a Sense of Normalcy Mitigated Potential Risk Factors Related to Runaway Decisions

A fourth key theme emerging from the interviews with youth centered around the importance of supports, structures, and contexts of the foster care placements themselves that may have abated the risk of youth running away. Youth accounts of *supportive* placements extended beyond the provision of basic needs and depicted how they helped youth experience a sense of belonging and normalcy. First, meaningful inclusion of youth voice in their foster care placements (e.g. placement selection, placement rules and privileges, etc.) promoted placement stability while a lack of youth voice in these daily care decisions promoted instability. One youth expressed a strong urge to feel heard and described running away as way to communicate a message, stating “I didn’t run, because I thought I was funny. I didn’t run it because I wanted to. I didn’t run because I think it’s fun. I ran to make a point, and they didn’t listen to me.” Other youth shared instances of trying to make their voices heard to improve their circumstances, and subsequently reported feeling additional trauma and powerlessness as a result.

For example, one youth shared that “when people [youth] tell [caseworkers] that the home’s not going well, to force them to stay there where they don’t fit in where they don’t feel loved, this causes more trauma than was already going to happen.” Another youth shared feelings of powerlessness, stating “when I really needed them, they weren’t there. And then when I really do not want them in my life anymore, they’re still here.” Youth consistently felt they were at the mercy of the system and that their voices, needs, and priorities were inconsequential to the care they receive from the child welfare system.

Second, the placement fit characterized by alignment between youth and the caregivers, and youth perceptions of belonging and familial support, contributed to decisions to remain in foster care or run away. One youth’s experience of feeling a sense of belonging and familial support within their placement demonstrates the stabilizing effect it had on their placement experience:

I’m in a really nice, loving family… I haven’t felt this comfortable with, like with anybody. They support me for whatever I want to do. They listen and they understand. They also don’t try to force their religion or their beliefs onto me. They just try to guide me through.

Conversely, another youth described how a lack of support may have worsened their trauma while in foster care and contributed to their decision to run away: “Before this family, foster care was hell every day. It didn’t matter which home they would have put us in, even if they were good, like they were just not fit for us.” As illustrated in these examples, unconditional regard was protective, mitigating additional runaway episodes. Whereas lack of support in foster homes contributed to additional episodes.

Additionally, several youth described advocating for themselves to caseworkers to improve the fit of their placements. For example, one youth shared they “tried to tell my caseworker that I can’t really, I don’t feel comfortable in my situation. But I can’t move.” One youth described running away as a desperate solution to an under resourced system that is unable to move youth who report poor experiences due to lack of suitable alternative placements. This may be exacerbated by practice decisions that have historically restricted relative and kin placements:

I guess for me, [not] fitting in was [making] me feel bad…Because I was bisexual or just didn’t feel connected with the mom or dad… for me it didn’t really go anywhere. I didn’t want to say that because I didn’t want to hurt their feelings. But I gave multiple hints to my caseworkers, and like this is not a good home for me, and I just don’t fit in I told the counselors, and they’d say well you have no other place to go. So, I would act up to be out of there.

This issue is impacted by both worker competency in centering the youth in decision-making and by system constraints and inability to move youth freely as required to adequately meet their needs and match youth closely to families.

Of particular note, several youth discussed experiences of differential treatment and lack of equity between foster and biological children in foster home placements that contributed to poor fit and decisions to run away. The differential treatment of foster youth took several forms. One youth explained how the foster parents in one placement required foster youth to perform household chores and duties not required of biological children:

In one of my homes, they didn’t even treat us like they should have. They treated us like dogs basically. Like we cooked, we cleaned, we did everything for them. And they let their own children, their biological children do whatever they wanted to.

Additionally, two youth discussed how differential treatment between foster children and biological affected them. One youth reflected on unmet social-emotional needs resulting from differential treatment: “It made us mad and hurt because we just wanted to be loved and have a home.” In a similar anecdote, another youth described emotional abuse with early experiences in foster care: “…when I first went in, the first like three of four foster homes were pretty rude and abusive, due to the fact that most foster parents favor their own kids.” In two cases, youth recounted experiencing physical abuse from biological children that went unaddressed by the caregivers. One youth described being bit by one of the biological children and recalling that “everybody in the house said ‘nope, that didn’t happen’.” Another youth remembered being “attacked by a kid because I was eating food that he wanted, even though I was given permission to eat it.” And yet another youth recounted experiencing overt racism in a foster home: “she [foster mother] would treat us different from our color of children. If we were lighter skin or white, she would treat us different than the people that were dark, dark skinned.” Across all of these examples, what is consistent is that youth did not feel valued or cared for in their placements and felt powerless to change their circumstances.

Despite these experiences that contributed to decisions to run away from foster care, youth also discussed several viewpoints and experiences with high quality and supportive placements. These experiences served to increase youth stability and promoted youths’ development, providing youth opportunities to pursue activities of self-actualization. Several youth offered insights into the benefit of supportive and high-quality placements centered around healthy communications, growing bonds with foster families, and the development of life skills critical to transition to adulthood. One youth described the dedication and support of their current placement and the development of familial bonds that have grown as a result:

They don’t give up on me whenever things do get hard. The fact that they sit there and actually talk to me and calm me down… Honestly, at this point, I think of them more as parents than just somebody I’m with for right now… I think of them more as family than I do a temporary placement.

Another youth discussed experiencing quality communication and positive discipline as a significant support in their current placement as opposed to punitive corrective actions they experienced before: “They don’t punish kids. They discipline kids if they think something is going on or if something’s wrong. They will talk to the kid, like ‘Hey, I’m just kind of worried. This is what I think is happening’.” One youth, who is also a parent, discussed the impact her current placement had on improving her own development of parenting skills and sense of competence. She described her growing sense of self-efficacy and confidence, recounting: “I’ve just been feeling like, okay, I can do this… I can be that mom that can be like going to school and getting my stuff together. I’m taking care of my baby and all of this stuff… it’s [current foster home] been a blessing.” This youth attributed her success to the support she received from her placement, sharing:

The people that I count on the most at this point is probably [foster dad] and [foster mom]. Because they have helped me through stuff. I’ve honestly thrived since I’ve been here. Because when I first got here, I struggled a little bit. I used to skip school, I ran away a few times, I did drugs, I did stuff that I wasn’t supposed to do. And now that I feel like as time has slowly gone by, I feel like I’ve actually thrived, because I’m not doing that stuff anymore.

When youth felt loved, supported, and heard, they consistently reported positive impacts on their lives, including increased stability.

#### System Shortcomings Fail to Protect Against Risk of Decisions to Run Away

The final theme emerging from qualitative interviews with youth involved a series of system shortcomings that may contribute to the risk of running away. Youth lamented a lack of individualized services and supports that involved them directly in service planning and accounted for their unique needs and context. Specifically, youth expressed a desire to be heard and trusted but reported not actually experiencing this in their interactions with agency and system staff. As one youth put it, “I wish that they weren’t like, behind a phone telling me what I got to do… You know, I wish that they would trust me more.” Another youth expressed a lack of connection or relationships with service providers as a contributing factor they weighed in decisions to run away, stating: “I just wanted to get away from all the services because I honestly hated everybody there. Everybody.” Conversely, when youth felt heard and supported by their caseworkers, they reported a greater sense of psychological safety. For example, one youth shared the following about their relationship with their current caseworker:

It’s a good relationship. I feel like I can go to her [caseworker] and I can tell her anything. And she’d be supportive. And she wouldn’t be really mad at me. And she just, she just be there for me if I needed her, honestly.

However, even when youth were able to develop connections with service providers, challenges related to staff turnover often negated the benefits associated with these connections.

One youth explained the trust and vulnerability required to divulge sensitive and personal information to a caseworker and the subsequent damage to relationships that results from a high degree of staff turnover. In this instance, the youth shared the difficulty of coming out and disclosing their sexual identity to a staff member for the first time who then left the position abruptly:

When they leave… I’m lost like I have nobody else… She was the first person I came out to, but she quit without even saying goodbye or me knowing… It took me like a week to have the courage to tell her that, but nope, she left three days later.

In some cases, case workers were the most stable force in youths’ lives despite frequent turnover. Many youth reported such frequent worker turnover that they routinely could not name their caseworkers. Many youth asked “which one” when asked to describe their relationships with staff, with one youth stating “I’ve had so many [workers] I really don’t even care anymore.” Abrupt changes without appropriate closure processes and support for youth created further conditions that promoted decisions to run away.

Beyond turnover, staff issues impacted youth experiences in foster care and decisions to run away. Time and again, youth described relationships and encounters with child welfare staff that were characterized by distrust, lack of clear communication and follow through, adversarial and uncompromising encounters, and in some cases, additional trauma. One youth reported remembering a caseworker who told them they “could never love anybody.” This youth reported this encounter as “the most messed up thing you could say” and as something that “has really stuck with me til this day.” This youth compared this encounter to “a slap in the face’ from a trusted person who “hurt me, my feelings.” Another youth reported being unable to rely on their worker to follow through.

They’re not gonna listen. I mean, I’ve been trying to get my driver’s ed test for a while or whatever and they always say, “Oh, yeah, we’ll get back to you on that.” But they still haven’t. It’s been like, I don’t know, maybe five months now since I turned 15. I mean, I get it. I know that they’re busy, but it’s just like eventually, you can just at least let me know, like, “Hey, I’m busy.” Or just like, anything. Really anything would help instead of, you know, saying, you’re going to come and do a house check, and you’re not.

These poor relationships between workers and youth were key factors in many youth decisions to run away from foster care.

Finally, building upon the importance of historical familial and relational connections identified in the first two themes of this analysis, system constraints keeping youth from connecting with their parents, siblings and other family members also emerged as a challenge. Specifically, poor visitation supports may have also contributed to youths’ decisions to run away. One youth shared having limited to no contact with their younger siblings because “their foster parents do not like me and my family so they do not allow us to have contact with them.” Reasons for lack of contact included system restrictions, worker decisions, geographic challenges, and real and perceived consequences. One youth shared being unable to have contact or “a conversation or something” with their father because he lost custody of the youth. Another youth reported visitation as a source of ongoing trauma, believing that “every time that I would do something bad, I was afraid that I was going to like, not get to see my parents again.” Another youth reported being unable to visit due to geographic limitations, compounded by their worker’s bias:

I don’t get to see any of them right now, because they are two hours away, and last time I seen them [sibling name] she stole money from me, and ever since then, [sibling name] my caseworker has not trusted my sister at all.

Workers’ control of youth access to family emerged from youth stories routinely, highlighting the potential need for standard procedures to eliminate subjectivity inherent in this decision making.

In part, due to the pervasive challenges limiting connection between youth and their families of origin, youth reported many missing or tenuous family connections. Many youth weren’t sure exactly where extended members of their families were, or when or if they might get to see them. For example, one youth who described knowledge and acceptance that he would not be reintegrated with his mother in a different state shared that despite this, he would “like to see her once or twice before I turn 18” to restore the relationship he was hoping to have upon exiting the foster care system. However, he perceived that this was not a possibility, assuming the state would not support purchasing “a whole ass plane ticket” but hoped maybe someday they could “meet halfway.” Study themes described the importance among youth of maintaining connections to members of their self-identified families of origin and sources of support. In the absence of system level supports for maintaining these connections as described in this theme, youth reported dire views of the future for their family connections “Without any kind of… without any contact, it might take me six years to even think about her [mother] being a big part of my life again, or a part.” This uncertainty was detrimental to youth stability.

## Discussion

This study was conducted to develop deeper understanding of runaway risk from a youth-centered lens to inform program innovations that will support youth placed in foster care, with a goal of increasing placement stability and reducing the incidence of runaway episodes. We used a mixed method approach with administrative data and youth qualitative interviews to identify predictors of youth absence from foster care. The findings of this study are intended to guide both the understanding of the issue of youth running away from foster care as well as inform practice and policy improvements that promote youth stability in care.

Specifically, we explored the following questions: (1) Is the population of youth in foster care placement with one agency in Kansas comprised of subpopulations of youth characterized by youth connections, youth health, critical life experiences, and youth system experiences; (2) If so, does subpopulation membership predict runaway from foster care placement; and (3) Do youth experience critical life experiences that serve as risk or protective factors that impact the likelihood of youth running away from foster care?

Results revealed that the population of youth in care with TFI who were ages 12 and older is comprised of four sub-populations characterized by these factors and experiences. Additionally, class membership, gender, number of siblings, and age are all significant predictors of runway behaviors. These results largely reflect literature previously discussed, which shows higher risk of runaway behavior among youth with substance abuse, conduct disorders, behavior problems, or mental health diagnoses (Nesmith, 2006; Lin, 2012; Courtney et al., 2005; Courtney & Zinn, 2009), and among older youth (Kim et al., 2015; Courtney & Wong, 1996; Courtney & Zinn, 2009; Courtney et al., 2005; Wulczyn, 2020; Lin, 2012; Nesmith, 2006). Finally, the findings of this study confirmed the theoretical and empirical importance of sibling relationships (Chor & Lou, 2021; Waid 2014).

Qualitative interviews with youth revealed five key themes related to youth life experiences that serve to mitigate or exacerbate youth decisions to run away from foster care placements. These themes highlight the critical nature of family connections as a mitigating factor supporting youth stability in foster care, along with youth, placement, and system factors that influenced youth decision-making related to running away. These findings are also consistent with previous literature that showed that youth with a permanency plan other than reunification were 89% more likely to run away (Nesmith, 2006), as were youth placed in congregate care (Courtney et al., 2005; Wulczyn, 2020; Courtney & Wong, 1996; Lin, 2012). Previous literature also suggests that youth who run away experienced more placements and more removals than those who did not run away (Kim et al., 2015; Pergamit & Ernst, 2011). This study adds to this body of literature reflecting the important mitigating role of family connections and familial belonging for youth. Together, these studies suggest that youth who are in situations with little familial connection, who have fewer familial resources to draw from, and who have had multiple disruptions to their familial connections, are more likely to run from their foster care placements.

While both the qualitative and quantitative findings shed some light on the factors that impact youth decision-making, triangulating findings across data sources helps us more deeply understand the phenomenon of youth runaway. Importantly, both the administrative data and youth interviews revealed key findings highlighting the importance of family connections as a protective factor for youth. Specifically, the subpopulation of youth most likely to experience runaway episodes is Class IV, or the *Low Stability and Low Environmental Connection* class. This class was characterized by multisystem involvement (i.e., child welfare and juvenile justice), and had low indicators of family stability and familial connection, including the highest probability of having a deceased or incarcerated parent or caregiver, lowest number of siblings, and lowest likelihood of being placed with siblings.

Paired with qualitative findings, we understand that youth with past runaway episodes often cite the lack of familial support and missing relationships as factors that influence their decisions whether to run from care, and whether to return to foster care from a runaway episode. Youth with few connections or limited access to connections reported low incentive not to run when faced with challenges in foster care placements. These youth also sought out supportive kin and non-relative kin relationships while on the run to meet their concrete and emotional needs. Finally, youth also reported returning to care specifically to mitigate the impact of their absence on loved ones. In sum, the primacy of familial relationships was evident in motivating youth decision-making related to placement stability. Leveraging these relationships to promote sustained familial connections for the youth that they can draw from when faced with challenging circumstances may serve to promote stability and mitigate decisions to run away from foster care. These findings are also consistent with the quantitative findings from a Kansas study of the predictors of permanency. That study found that connection to family via sibling placements was a statistically significant predictor of reunification, and conversely, not being placed with siblings was a predictor of adoption (Akin et al., 2011).

Importantly, Class IV was comprised primarily of older male youth who were the most racially diverse of all the subgroups, and youth in this subpopulation were more likely to be removed due to child behaviors. This finding reflects the cumulative impact of complex system, environmental, and personal circumstance, but is also likely indicative of a lifelong experience of oversurveillance by systems of power and oppressive and racist policy and practice that target Black and Brown families, resulting in these youth grouping in the highest risk class. This level of surveillance and the impact of complex stressors are also evident in other trends for Black and Brown youth in child welfare, with these youth being placed in congregate care settings at a higher rate than white youth (Branscum & Richards, 2022).

When paired with qualitative findings, it is also not surprising that this class experienced the highest likelihood of a runaway episode. While some youth reported making decisions to run away, many youth with runaway history reported making rash decisions triggered by harsh restrictions and challenges in foster care placements with belonging (e.g., overt and covert racism, inequity, harsh punishment, placement restrictions, lack of support, etc.) that they later regretted and understood did not solve their issue. However, youth routinely described difficulty with impulse control when triggered by their environment, a response common among adolescent boys in general, and youth with externalizing behavior disorders specifically. Thus, the high occurrence of runaway behavior among this group is not unexpected.

Additionally, though Class IV arose as the subpopulation of highest concern in regard to runaway risk, Classes II and III were not statistically significantly different from Class IV, indicating some elevated level of risk among these groups as well, in particular among Class III.

Class III, characterized as the *Environmental Stability/Low Community Connection but High Familial Connection* subpopulation is unique in the high level of placements among members of this group, low proximity to family, and high probability of health, mental health and developmental issues.

This class is also unique in its composition, consisting of more female than male youth and youth with a higher probability of removal due to parent and parenting related reasons (i.e., parental substance use, physical abuse, and neglect). Therefore, youth in this class have also likely experienced disruptions to familial connections including removal due to parent action or inaction, low proximity to family, and potential disruption to placement connections due to psychiatric hospitalizations. However, this class also has the highest level of family visits and thus may have slightly more familial resources to draw from. Therefore, despite complex challenges, members of this subpopulation are slightly less likely to run from their foster care placements than youth in Class IV. However, it is important to note that all youth in foster care, despite class membership, would benefit from changes in policy and practice that support maintenance of family connections as a primary goal of the system.

### Limitations

While this mixed methods study produced relevant findings to answer the research questions and inform the broader field about youth experiences with running away from foster care, it is important to elaborate on a few of the study’s limitations. Regarding the quantitative component of the study, analysis relied exclusively on an administrative dataset compiled by TFI primarily for ongoing case management, monitoring, and reporting purposes. Because of this, the low prevalence of some factors (i.e., LGBTQ identity) may have impacted the precision of the analysis and whether some factors arose as important. Despite this limitation, we were able to draw some conclusions about the importance of fit and belonging for populations with marginalized identities such as Black and Brown youth and youth identifying as LGBTQ. Furthermore, not all variables of interest were collected or represented in the exact manner intended for the research questions. There were likely confounding variables that were unable to be included during quantitative analyses that may otherwise influence latent class membership and the likelihood of runaway episodes. For example, variables such as kinship placement and racial and ethnic match between youth and foster care families would ideally be included. Because they were not included in the administrative data, they were not included in our quantitative analysis. While we accounted for these qualitatively, future studies should prioritize additional exploration of these areas and their impact on youth runaway behavior.

Furthermore, as previously described, proxy variables were created to minimize other limitations of the administrative dataset. While these proxy variables allow for the inclusion of critical information germane to these analyses, important information that may impact the findings of this study were not able to be captured. For example, while the proximity variable represents physical proximity and potential access to family and community connections (e.g., parents, teachers, coaches), proximity does not equate to connection or routine and supported visitation. Similarly, known participation in gang activity and violence is not exhaustive of the types of community violence to which youth may be exposed, and the sexual trauma variable captures commercial sexual exploitation, but does not measure prevalence of rape and sexual abuse. Finally, use of administrative data limited our ability to differentiate between types and rationales for running away. Thus, the mixed methods design of this study was critical to further mitigating these limitations.

Further, we recognize the class size imbalance as a limitation of this study. Though the 4-class solution emerged as the optimal solution, the class distribution was severely imbalanced, with Class I making up 83% of the sample and Classes III and IV each making up 2% of the sample. While we considered retaining the 3-class solution as the final model, careful examination of our findings supported our model selection. While the 3-class solution did reveal a class at high risk of running away, when we compared the 3-class and 4-class models, we identified distinct and substantively important differences that have implications for practice, policy, and research. For example, the 4-class model differentiated Classes III and IV based on biological sex and system experiences. The youth in Class III were largely female and had more hospitalizations while youth Class IV were largely male and had more juvenile justice experiences and child welfare system placements. Taken together, the evidence supported retention of the imbalanced model, but highlight a need to continue studying this issue. However, due to the small size of these classes, we encourage caution in generalizing the quantitative findings and suggest that future study should include a longitudinal sample as opposed to a cross-sectional youth-in-care sample to replicate and validate this model.

Concerning the qualitative component of the study, the interviews were limited to remote engagement of youth via telephone or virtually via Zoom teleconferencing. COVID-19 pandemic restrictions, technology requirements (e.g., phone, text, and data plans), and scheduling complexities to accommodate youths’ schooling and extra-curricular activities and communications between case workers, researchers, foster and residential placements, and youth all impeded the full depth of conversation that could occur during the interviews. Additionally, and perhaps most significant, while we obtained youth assent and encouraged youth to participate in interviews in a private space, youth did not always have access to separate, quiet, or private spaces to engage in the interviews. Multiple youth participants reported being in the presence of others during the interview, including one youth who participated while riding as a passenger in a vehicle with their foster parent. The interviewers took this limitation seriously and checked in often with youth to ensure they were willing to continue and offered opportunities to reschedule as it appeared that being around others influenced youths’ responses and engagement. Further, the member checking and co-interpretation webinar held with youth provided another opportunity to check in with participant youth to ensure findings accurately reflected their experiences, contributions, and intent. However, future qualitative investigations would benefit from administering interviews in a private, face-to-face setting, with additional follow-up.

Despite these challenges, the overall strength of the study’s design may reduce the impact of its limitations. The study utilized a person-centered methodological approach that amplifies youth voice. Conversations with youth yielded thoughtful and powerful narratives about their experiences, perspectives, and involvement with the child welfare system as well as the importance of establishing and maintaining strong relationships with caregivers and other family members that extend beyond providing or their basic needs. We further complemented the person-centered approach by pairing this approach with a quantitative, variable-centered analysis. Doing so presented opportunities to triangulate findings and ensured a robust and nuanced analysis (Kusurkar et al., 2021) despite other limitations.

### Implications for Practice, Policy, and Research

Practice, policy, and research implications, in the form of recommendations for systems change resulting from this study are provided in three key domains: (1) improving family visitation and maintenance of youth connections with self-identified family and non-relative kin; (2) supporting service approaches for youth that honor and amplify their voices, choices, and family connections; and (3) improving the quality of placements, and individualization of services. Within each of these domains, we recommend several considerations for improving policy and practice to support stable and safe placements for youth where they feel a sense of belonging, a voice in their care, and strong support for remaining connected to loved ones.

#### Improving Family Visitation

Key to ensuring youth have the connections necessary to support reintegration, permanency, and support across the life course, it is necessary that family visitation for youth in foster care be overhauled to ensure more frequent and extended visitation with family members identified by the youth (e.g., relative, kin, and non-relative kin). Further, this visitation should be sustained regardless of case plan goals, youth behavior, or parent compliance with case plan requirements and activities. The goal is to maintain ongoing and lasting family connections that will endure beyond the youths’ time in foster care. For example, when reflecting on this recommendation during co-interpretation, one youth shared his perception that he was to blame for his lack of family connection because visitations were cancelled due to his behavior. He further shared that his family also blamed him for these missed opportunities to connect, further compounding the disconnection for this youth.

This enhanced approach to visitation also has the added benefit of honoring youth perspectives and protecting youth from relationships they deem personally harmful. For example, one youth shared anger at being forced to hold visitation with her mother whose behavior she saw as harmful to her well-being. However, this stance was eclipsed by routine practice and the youth was required to attend visitation. Following along with this recommendation, youth in foster care would have an authentic opportunity to weigh in on all components of their visitation plans. Additionally, visitation would be provided with the least restrictive conditions necessary to maintain youths’ safety, only being restricted when actual rather than perceived threats to safety are present. It is also imperative that system players identify alternative means of leveraging “compliance” from youth and families that do not threaten key familial relationships.

#### Amplifying Youth Voice and Choice

Further, findings from this study highlight the importance of supporting permanency and stability approaches for youth that honor and amplify their voices and family connections. To achieve this goal, we recommend that foster care providers adopt a *shared parenting* model of practice, consider and adopt eligibility criteria for kinship placement that prioritize family and kin placements as a first consideration, minimizes restrictions that prevent youth placements with family and kin, and continues to embrace avenues for integrating the youth’s needs, preferences, and wishes into case planning.

Though shared parenting is a concept that has emerged in the literature and in practice in recent years, it has not previously been well-defined. Through collaboration with the Improving Child Welfare through Investing in Families (ICWIF) grant cluster supported by the U.S. Department of Health and Human Services Children’s Bureau, shared parenting has been defined as a practice intended to support family connection, quality care, and ultimately, successful reintegration. This is achieved through an intentional practice of developing relationships among these partners that are built on trust and support but do not replace the parent. Rather an alliance is established between workers, caregivers, and parents so that parents can continue to support and protect their children and enhance family well-being while their child is in foster care. ​These relationships include different roles, responsibilities, and tasks, but the overarching goals remain the same across cases: ensure clear communication, work in partnership as a united front, create normalcy for parents, and change individual attitudes and organizational culture to embrace shared parenting principles. A shared parenting approach to child welfare ensures that parents and kin continue to have a meaningful role in the youth’s life, ensuring this connection and resource is intact whether the youth returns home or not.

Further, we recommend that foster care provider and regulatory agencies consider reviewing and adopting eligibility criteria for kinship placement that minimizes restrictions that inhibit routine use of kinship care. While current policy in Kansas is broad in order to support use of practice wisdom to guide decision making, in reality, if kin are identified, kinship placements are often restricted by the size or condition of the kin home, inability of kin caregivers to afford caring for the child, past criminal offenses, family pet breeds, and substance use, among other factors (Byers et al., 2022). Removing these barriers to placement when the environment does not pose an immediate threat to safety ensures that youth can be placed with their family members and non-relative kin and maintain connection and normalcy while out of the home. Adapting policy and practice to ensure these homes receive the same financial and concrete support as traditional foster homes is also essential to successful use of kin placements.

Kansas child welfare administrators are actively exploring new kinship permanency options (e.g., SOUL Family), supporting new kinship support and navigation programs through statewide, federal, and local initiatives (e.g., Kids2 Kin legal support, Kansas Invests in Families, kinship navigation grants). Child welfare advocates have also realized policy improvement for kinship families in the 2023 legislative session with kinship care rates being raised to 70% of the foster care daily payment rate. Additionally, the federal government is currently proposing new policy language allowing states to adopt more flexible licensing standards for kinship care, helping to mitigate barriers to kinship care. Together, these efforts begin to provide the support and infrastructure necessary for a robust kinship program that helps keep families together. The empirical evidence derived from this study highlights the critical need for initiatives of this type to support youth safety, permanency, and well-being through connection to kin.

It is essential that youth continue to have voice and choice in the placement decisions made, including where and with whom they live, and as developmentally appropriate, the rules, restrictions, and privileges of their placement. Together, these actions will improve overall fit of placements for youth and promote a sense of normalcy, belonging, and sustained family connection.

#### Improving Placements and Services

Finally, we recommend improvement to the quality of placements, including ensuring high degree of “fit” and alignment of youth needs and identities with placement characteristics. Ensuring fit serves to promote a sense of belonging. However, this must also be paired with more robust oversight of placement conditions and treatment, frequent and effective communication, responsiveness to conflicts that may cause disruption, and elimination of unnecessary placement restrictions based solely in state liability.

Assessment and consideration of fit is especially key for populations such as Black and Brown youth who are routinely placed in families with different racial and ethnic identities than their own (Branscum & Richards, 2022) and for youth who identify with LGBTQ sexual identities. The lack of fit often inherent in placements where these identities are not carefully considered can result in further trauma for youth in care. Assessment of youth needs and characteristics should be carefully considered and accounted for in making placements to ensure increased normalcy and long-term placement stability.

Further, increased individualization of services to meet the needs of youth are essential. Youth routinely reported one size fits all approaches and required services they felt were unnecessary or not useful. Service plans should be unique, developed together with the case team, including the youth, parent, and caregiver, and should meet the parent and youth where they are. To meet this goal, we recommend that state child welfare agencies examine and reconsider regulations governing trained and licensed foster families that are based in state liability and restrict normalcy and distinguish foster youth from other youth in households. Reviewing and relaxing placement rules to allow caregivers to use their parental judgement may result in a more normalized experience for youth where they are allowed privileges and activities (e.g., overnight sleepovers, cell phone use, trampolines) based on their developmental readiness and behavior as determined by their caregiver, as is typical for adolescent youth not in foster care. Allowing foster parents and youth more autonomy to design their lives may minimize conflicts requiring further intervention from workers, disruption, and runaway episodes. Finally, we recommend screening youth for risk for runaway and individualizing wraparound supports and enhanced placements (e.g., Therapeutic Foster Care) initially and ongoing to ensure the supports needed for healthy youth development are present, including protection from runaway events.

## Conclusion

This study of youth in foster care seeking understanding of risk and protective factors that impact youth decisions to run away from foster care leaves us with two key takeaways. First, maintenance of youth-identified familial and kinship bonds, youth autonomy, and supportive services that honor family bonds, are necessary to promote a sense of belonging and support that ensures youth placement stability and ultimately permanency. Second, system-level practice and policy should support connection and maintenance of these bonds as well as development of new bonds in quality placements as a primary goal of the system from initial contact throughout the duration of the case. This support must be maintained regardless of the case plan goal to ensure that youth leave care with a robust network of familial connections and relational permanence, regardless of their legal permanence status.
